# A Bayesian latent class analysis for whole-genome association analyses: an illustration using the GAW15 simulated rheumatoid arthritis dense scan data

**DOI:** 10.1186/1753-6561-1-s1-s112

**Published:** 2007-12-18

**Authors:** Fredrick R Schumacher, Peter Kraft

**Affiliations:** 1 Department of Epidemiology, Harvard School of Public Health, Kresge Building, 677 Huntington Avenue, Boston, Massachusetts 02115, USA; 2 Program in Molecular and Genetic Epidemiology, Harvard School of Public Health, Kresge Building, 677 Huntington Avenue, Boston, Massachusetts 02115, USA

## Abstract

Although rheumatoid arthritis, a chronic and inflammatory disease affecting numerous adults, has a complex genetic component involving the human leukocyte antigen region, additional genomic regions most likely affects susceptibility. Whole-genome scans may assist in identifying these additional candidate regions, but a large number of false-positives are likely to occur using traditional statistical methods. Therefore, novel statistical approaches are needed. Here, we used a single replicate from the Genetic Analysis Workshop 15 simulated data to assess for marker-disease associations in 1500 rheumatoid arthritis cases and 2000 controls on chromosome 6. The statistical methods included a maximum-likelihood estimation approach and a novel Bayesian latent class analysis. The Bayesian analysis "borrows strength" from multiple loci to estimate association parameters and can incorporate differences across loci in the prior probability of association. Because of this, we hypothesized that the Bayesian analysis might be better able to detect true associations while minimizing false positives. The Bayesian posterior means for the log alleleic odds ratios were less variable than the maximum likelihood estimates, but the posterior probabilities were not as good as the simple *p*-values in distinguishing a signal from a non-signal. Overall, Bayesian latent class analyses provided no obvious improvement over maximum-likelihood estimation. However, our results may not be able to be generalized due to the large effect simulated in the human leukocyte antigen-DR locus.

## Background

Rheumatoid arthritis (RA) is the most common cause of inflammatory polyarthritis in adults [1]. This chronic, inflammatory disease has a complex genetic component involving the human leukocyte antigen (HLA) region. For nearly two decades the association between the HLA region and RA has been known and confirmed in numerous population studies [2]. The HLA associations are extremely complex and their exact biologic role in RA is unknown. Furthermore, family and twin studies have indicated non-HLA genes may play an important role in RA.

Advancements in genotyping technology have facilitated the ability to generate large amounts of genetic data. The large numbers of single-nucleotide polymorphisms (SNPs) genotyped by whole-genome scans may overwhelm conventional statistical approaches such as maximum-likelihood estimation (MLE). A few novel statistical approaches reducing the dimensionality of large data sets and detecting the structural relationship between variables have been described elsewhere [3-5]. Simple association tests may produce a large number of false positives; therefore newer statistical approaches are needed to incorporate known information on disease etiology, thus reducing the potential for these false-positive associations. Bayesian analysis might be better able to detect true associations while minimizing false positives, because it can "borrow strength" from multiple loci to estimate association parameters and it can incorporate differences across loci in the prior probability of association. Appropriate Bayesian analysis should also reduce parameter estimate variability, similar to penalized regression methods such as ridge regression and the lasso (which are themselves special cases of Bayesian analysis [6]). As a methodological exercise, we contrast a novel Bayesian latent class analysis with MLE in a simulated data set of RA for chromosome 6 markers from the Genetic Analysis Workshop 15 (GAW 15 Problem 3). Although our analysis is restricted to a subset of the data that would be available from a genome-wide scan, in principle, our method could be applied to a whole-genome scan.

## Methods

We randomly selected one RA case from the affected-sibling pair (ASP) in the first replicate of the GAW15 simulated data for Problem 3. After selecting all of the controls, our final data set included 1500 cases and 2000 controls. In order to ensure a significant finding we reviewed the answers to the simulated data prior to our analyses. Since the strongest signal for RA was simulated to the HLA region on chromosome 6, we limited our analyses to the dense genotyping for chromosome six. In total 17,820 SNPs were simulated on chromosome 6, yielding an average inter-marking spacing of 9586 base pairs. This corresponds to the density one would expect from a genome-wide 300,000 K SNP set.

### Model

For each SNP *i*, we modeled the 2 × 2 allele-by-disease status table using a hypergeometric likelihood with OR = exp[*β*_*i*_] [7]. The prior on the log allelic odds ratio *β*_*i *_is a mixture of point mass at 0 with a distribution of N(*μ*_*j*_, *σ*_*j*_), where *j *= 1,.., *J*, and *J *is the number of non-null classes. For example,

$$\beta_{i}=0+\sum_{j=1,...J}1[X_{i}=j]\beta_{ij}$$

where *β*_*ij *_is ~N(*μ*_*j*_, *σ*_*j*_) and *X*_*i *_is binomial or trinomial (0,.., *J*) with probabilities

$$(1-\sum_{\text{i}=1,..,\text{J}}\pi_{\text{i}},\pi_{1},..,\pi_{\text{J}}).$$

We considered two ways to separate the markers into associated (non-null, *X *> 0) and non-associated (null, *X *= 0) classes. First, we naively assume all non-null loci are derived from the same distribution (*J *= 1). Second, we assume some markers are positively associated with the outcome, i.e., OR > 1, and others are inversely associated with the outcome, i.e., OR < 1 (*J *= 2).

A vast majority of the disease-marker associations will be null, so we used conjugate priors to update *μ*_*j *_and *σ*_*j*_. Conjugate priors are helpful when the number of non-null loci is small and they may provide information distinguishing between classes, i.e., OR < 1 or OR > 1, for the model where *J *= 2. In principle non-identifiability is a problem; however, by putting very small prior probabilities on identical alternative parameterizations we may avoid this issue [8]. The conjugate priors for *μ*_*j *_and *σ*_*j *_were *μ*_*j*_|*σ*_*j*_~ *N*(*μ*_0_, *σ*_*j*_/*κ*_0_) and *σ*_*j *_~ Inv-*χ*^2^(*σ*_*j*_, *ν*_0_). The hyperparameters we used were,

for *J *= 1

*μ*_0 _= log(2), *κ*_0 _= 5, *ν*_0 _= 5, *σ*_0_^2 ^= log(2)/2,

and for *J *= 2

*μ*_10 _= log(2), *κ*_10 _= 5, *ν*_10 _= 5, *σ*_10_^2 ^= log(2)/2,

*μ*_20 _= -log(2), *κ*_20 _= 5, *ν*_20 _= 5, *σ*_20_^2 ^= log(2)/2.

We put Dirichlet priors on *π *= (*π*_0_,.., *π*_*J*_). For example, when *J *= 1, *π *~Dirichlet (1, 999). To account for differences in prior probability of association, we also varied Dirichlet hyperparameters across regions. We selected three candidate regions with identical, high prior probability of association after reviewing the answers for the simulated data and performing a literature search on RA. The HLA region, where the causal SNP was simulated, and two upstream regions from the literature search were up-weighted. The two upstream regions contained several genes, including *WISP3 *and *VIP*, with a potential biologic role in RA [9,10]. We fixed the ratio of prior odds of association between candidate regions and non-candidate regions at ≈ 53. For example, when J = 1 we set *π *~Dirichlet(1, 999) in non-candidate regions and *π *~Dirichlet(0.25, 4.75) in candidate regions. In the interest of time and to reduce the computational burden we chose every fifth SNP from the dense data on chromosome 6, for a total of 3564 markers. Of these, 62 were in the HLA region and 22 in each of the two upstream regions. In principle the latent class analysis has no limit as to the number of SNPs that can be analyzed, and given optimized code, more computing resources and more time, an analysis of 300,000–500,000 markers from a genome-wide scan is feasible. Each analysis presented here took approximately 12 hours on three nodes (each with two 3.2 GHz CPUs), so a scan with 300,000 markers would take less than five days on a 30-node cluster.

### Model fitting

We used three parallel Gibbs sampling chains with 3000 iterations each in order to fit the model. The parameters *μ*_*j*_, *σ*_*j*_, and *π*_*j *_could be updated by directly sampling from their conditional posterior distributions. The parameters *β *and *X *were simultaneously updated using the Metropolis-Hastings algorithm.

## Results and discussion

**Figure 1:**
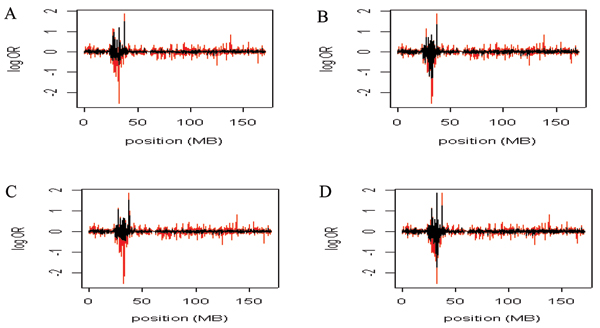
**The beta estimates from the MLE and Bayes models**. The Bayes (black) and MLE (red) beta estimates for model *J *= 1 and *J *= 2. The Bayesian estimates are the mean of the posterior. The x-axis is the marker position and the y-axis is the log OR. Panel A, model 1; Panel B, model 2; Panel C, model 1, non-associated/associated weights; Panel D, model 2, non-associated/associated weights.

**Figure 2:**
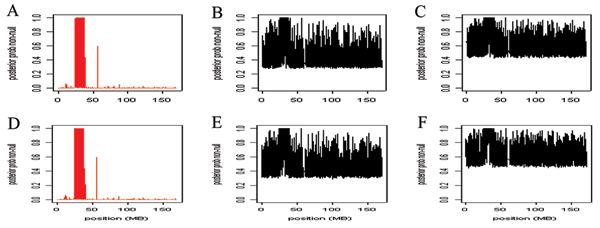
**The probabilities for a true-positive result using the MLE and Bayes models**. The posterior probabilities of true-positive results for priors plotted against the marker position. The posterior probabilities are transformed frequent *p*-values, 0.0001/(*p *+ 0.0001). The red plots are from the MLE estimates and the black plots are from the Bayesian estimates. Panel A, MLE estimates; Panel B, model 1, Bayesian estimates; Panel C, model 2, Bayesian estimates; Panel D, MLE estimates; Panel E, model 1, Bayesian estimates, non-associated/associated weights; Panel F, model 2, Bayesian estimates, non-associated/associated weights.

The posterior means for the log allelic odds ratios are presented by marker position for the MLE (red) and Bayes (black) models in Figure 1. Parameter distributions were similar across chains, so all models appeared to converge (results not shown). Figure 1A and 1B show the two Bayes models without weighted priors. The Bayes estimates in Figure 1C and 1D are from the models in which the priors were either weighted as associated (candidates) or non-associated (non-candidates). Across the panels the peak lies within the HLA region. However, the log allelic odds ratio (*β*) estimates from MLE are more dispersed than the Bayes estimates. In Figure 2 the posterior probabilities of true-positive results are presented for the MLE model (red) and Bayes models *J *= 1 and *J *= 2 (black). The top panels represent the model without weights and the bottom panel represents the model with weights. The probabilities for a true-positive result are nearly evenly dispersed across the markers for the Bayes estimates, whereas the MLE probabilities peak near the HLA region and decrease substantially elsewhere (Fig. 2).

The average estimated log allelic odds ratios varied slightly across the MLE and Bayes models (Table 1). The average *β *values are given for four regions and a combined region. Although the estimates were nearly equal for the non-candidate region, the average *β *values for the HLA region differed slightly. The Bayesian models produced nearly equal values (-0.305 with priors and -0.329 without priors), whereas the MLE average was greater (-0.575). For candidate region 2, the estimates were nearly equal across the models, but the average *β *values differed for candidate region 1. The average *β *from the MLE model was greater in candidate region 1.

**Table 1:** Average beta estimates from the MLE and Bayes models across candidate and non-candidate regions

Model	Non-candidate	HLA	Region 1	Region 2	HLA + Region 1 + Region 2
MLE^a^	-0.005	-0.575	0.014	0.01	-0.331
Without priors^b^	-0.004	-0.329	0	0.005	-0.191
With priors^b^	-0.004	-0.305	0.001	0.007	-0.177

^a^Maximum likelihood estimates

^b^Bayesian estimates. With priors is weighted and without priors is unweighted.

## Conclusion

We developed and implemented a Bayesian latent class analysis because we hypothesized that by "borrowing strength" across multiple loci and incorporating prior probabilities of association, such an analysis might be more sensitive and specific than *p*-values from maximum-likelihood based tests. We applied this latent class analysis to the GAW15 simulated chromosome 6 data, but found that the latent class models provided no obvious improvement over MLE. However, our overall results may not be generalizable due to the large simulated effect in the HLA-DR locus.

As can be seen in Figures 1 and 2, the Bayesian posterior means for *β *is less variable than the MLE. This suggests that if researchers are choosing markers to follow up on the basis of estimated effect size, some sort of smoothing procedure could be useful. MLEs for rare SNPs may be very unstable. Smoothing MLEs to a group mean will account for differences in information across SNPs and hence could reduce the false-positive report probability [11].

On the other hand, the posterior probability of association Pr(*X *> 0) from the latent class analyses was not as good as simple *p*-values distinguishing a signal from a non-signal. Negative consequences, although minor, occur when up-weighting regions that are not true candidates (Table 1). In the GAW15 simulated data set, when the HLA signal was extremely large, the weighting of candidate regions did not appear helpful.

There are clearly drawbacks to the latent class approach as we have implemented it. The posterior probability of belonging to a non-null class, Pr(*X *≠ 0), is much too large for a majority of the loci. Setting *f*(*β*) equal to point mass at 0 may be too stringent. One potential solution is to allow for some noise to distinguish signals near, but not directly at, zero from true effects. Additionally, it appears the priors are overwhelmed by the large amount of data (loci). Because a majority of the loci are likely null, the prior sample size (e.g., *κ*_0 _or the absolute magnitude of *π*_*j*_) could be increased. Other drawbacks to our implementation include the facts that the ratio of priors comparing candidate regions to non-candidate regions is fixed, and only loci in regions sharing the same prior contribute to estimating the posterior odds of association in those regions. A hierarchical Bayesian approach may be more appropriate because it estimates both baseline and relative odds of association from the data, rather than fixing them [12]. By not fixing the prior odds in non-candidate regions, the hierarchical Bayesian approach may also be more sensitive to true associations in unexpected regions (e.g., regions with little biologic annotation).

## Competing interests

The author(s) declare that they have no competing interests.
